# Quantitative threefold allele-specific PCR (QuanTAS-PCR) for highly sensitive *JAK2* V617F mutant allele detection

**DOI:** 10.1186/1471-2407-13-206

**Published:** 2013-04-24

**Authors:** Giada V Zapparoli, Robert N Jorissen, Chelsee A Hewitt, Michelle McBean, David A Westerman, Alexander Dobrovic

**Affiliations:** 1 Department of Pathology, Peter MacCallum Cancer Centre, St Andrews Place, East Melbourne, Victoria, 3002, Australia; 2 Walter and Eliza Hall Institute of Medical Research, Parkville, 3052, Australia; 3 Department of Medical Biology, University of Melbourne, Parkville, Australia; 4 Division of Cancer Medicine, Peter MacCallum Cancer Centre, St Andrews Place, East Melbourne, Victoria, 3002, Australia; 5 Department of Pathology, University of Melbourne, Parkville, Victoria, 3010, Australia; 6 Sir Peter MacCallum Department of Oncology, University of Melbourne, Parkville, Victoria, 3010, Australia; 7 Molecular Pathology Research & Development, Peter MacCallum Cancer Centre, St Andrews Place, East Melbourne, Victoria, 3002, Australia

**Keywords:** Mutation detection, Myeloproliferative neoplasms, Minimal residual disease, qPCR, Real time PCR, *JAK2* V617F, *BRAF* V600E, *EGFR* T790M

## Abstract

**Background:**

The *JAK2* V617F mutation is the most frequent somatic change in myeloproliferative neoplasms, making it an important tumour-specific marker for diagnostic purposes and for the detection of minimal residual disease. Sensitive quantitative assays are required for both applications, particularly for the monitoring of minimal residual disease, which requires not only high sensitivity but also very high specificity.

**Methods:**

We developed a highly sensitive probe-free quantitative mutant-allele detection method, Quantitative Threefold Allele-Specific PCR (QuanTAS-PCR), that is performed in a closed-tube system, thus eliminating the manipulation of PCR products. QuantTAS-PCR uses a threefold approach to ensure allele-specific amplification of the mutant sequence: (i) a mutant allele-specific primer, (ii) a 3′dideoxy blocker to suppress false-positive amplification from the wild-type template and (iii) a PCR specificity enhancer, also to suppress false-positive amplification from the wild-type template. Mutant alleles were quantified relative to exon 9 of *JAK2*.

**Results:**

We showed that the addition of the 3′dideoxy blocker suppressed but did not eliminate false-positive amplification from the wild-type template. However, the addition of the PCR specificity enhancer near eliminated false-positive amplification from the wild-type allele. Further discrimination between true and false positives was enabled by using the quantification cycle (Cq) value of a single mutant template as a cut-off point, thus enabling robust distinction between true and false positives. As 10,000 *JAK2* templates were used per replicate, the assay had a sensitivity of 1/10^-4^ per replicate. Greater sensitivity could be reached by increasing the number of replicates analysed. Variation in replicates when low mutant-allele templates were present necessitated the use of a statistics-based approach to estimate the load of mutant *JAK2* copies. QuanTAS-PCR showed comparable quantitative results when validated against a commercial assay.

**Conclusions:**

QuanTAS-PCR is a simple, cost-efficient, closed-tube method for *JAK2* V617F mutation quantification that can detect very low levels of the mutant allele, thus enabling analysis of minimal residual disease. The approach can be extended to the detection of other recurrent single nucleotide somatic changes in cancer.

## Background

Myeloproliferative neoplasms (MPN) are clonal hematopoietic stem cell malignancies comprising several diverse pathologies. The 2008 World Health Organization (WHO) classification incorporated *JAK2* V617F mutation status into the diagnosis of BCR-ABL negative MPN [1]. This mutation is the most frequent somatic change in MPN, occurring in over 95% of patients with polycythemia vera (PV) and in 50% of patients with essential thrombocythemia (ET) or primary myelofibrosis (PMF) [2]. The valine to phenylalanine substitution at amino acid 617 causes a disruption of the auto-inhibitory JH2 domain of JAK2, leading to constitutive activation of the JAK2 tyrosine kinase activity and a consequent loss of control in cell proliferation and growth [3,4]. Small-molecule inhibitors targeting JAK2-driven cancers have recently entered clinical trials [5,6].

The *JAK2* V617F change results from the c.1849G > T point mutation in exon 14 of the *JAK2* gene (COSMIC ID: COSM12600). Numerous detection methods for the *JAK2* c.1849G > T mutation have been published including, restriction fragment length polymorphism analysis, Sanger sequencing, pyrosequencing, amplification refractory mutation system, allele-competitive blocker PCR, and melt curve analysis or high resolution melting (HRM) [7-20].

For the detection of minimal residual disease, highly sensitive quantitative assays are required. Several quantitative PCR methods based on mutation-specific primers or probes or LNA-modified oligonucleotides have been developed [21-27]. These range in sensitivity from medium (0.1-1% mutant alleles) to high (< 0.1% mutant alleles). Less sensitive assays (with a detection limit of 1-3% mutant alleles) are useful for disease diagnosis [28].

In the minimal residual disease context, the detection of low levels of the mutation can be challenging, especially due to the occurrence of false positives. Thus, accurate and sensitive V617F testing of treated MPN patient samples needs to be performed using assays that minimise or at least enable the recognition of false positives. For these purposes, we developed a new highly sensitive assay, based on allele-specific PCR, which is able to specifically and efficiently suppress amplification of the wild-type allele.

The method that we developed, QuanTAS-PCR, is a probe-free quantitative PCR method based on a threefold approach to ensure specific amplification of the mutant *JAK2* allele: (i) the use of allele-specific primers to amplify the mutant allele (sometimes known as allele-specific PCR or ARMS), combined with (ii) the use of a non-extendible dideoxy oligonucleotide complementary to the wild-type allele and known as a blocker, and (iii) the use of a PCR specificity enhancer. By combining these three measures, we achieved high analytical sensitivity (one single mutant allele per well) coupled with high analytical specificity, providing a robust quantitative assay.

## Methods

### Patients and controls

This study was covered by an approval from the Peter MacCallum Cancer Centre Ethics Committee (project number 03/90). DNA from 27 peripheral blood samples and 11 bone marrow aspirates of patients with a suspected diagnosis of myeloproliferative neoplasms had originally been used for diagnostic testing using our previously reported *JAK2* assay [17]. Normal controls included thirteen anonymised blood samples obtained from the Australian Red Cross Blood Service as well as an additional blood sample taken under informed consent. The human erythroblast leukemia cell line HEL, which bears a homozygous *JAK2* V617F mutation, was used as a source of 100% mutant DNA. The human promyelocytic leukemia cell line HL-60 was used as a source of 100% wild-type DNA.

### DNA extraction

DNA was extracted using either the DNeasy blood and tissue kit (Qiagen, Hilden, Germany) or the Wizard Genomic DNA Purification kit (Promega, Madison, WI) as per the manufacturer’s instructions. DNA quantification was performed using the Qubit dsDNA HS Assay kit and the Qubit 2.0 Fluorometer (Life Technologies, Carlsbad, CA). The Qubit readings were used as a guideline for dilution of the DNA samples.

### Dilution series for mutant allele quantification

A set of mutant allele dilutions was prepared by mixing quantification cycle (Cq) normalised HEL DNA (MUT) which harbors the mutant allele only, and HL-60 DNA (WT), which harbors the wild-type allele only. In earlier experiments, the HEL DNA (MUT) had been normalised for their *JAK2* copy number, based on their amplification, and mixed with genomic DNA extracted from a healthy blood donor (WT). Each MUT/WT mix was made to a final DNA concentration of 16.5 ng/μl, which corresponds to a total number of 5,000 *JAK2* copies/μl.

The number of *JAK2* copies/μl was calculated on the basis that one diploid human cell contains approximately 6.6 pg of DNA and therefore 33 ng human genomic DNA contains approximately 10,000 copies of each diploid gene. The mixes contained a decreasing proportion of the mutant allele relative to the wild-type allele, as follows: 30%, 10%, 3%, 1%, 0.3%, 0.1%, 0.03% and 0.01%. The 100% mutant and the 100% wild-type control DNA samples were included in each PCR. For quality control, the total *JAK2* copy number of each MUT/WT mix was tested by running the *JAK2* exon 9 PCR, using LinRegPCR 12.5 software [28-30] which can be downloaded at http://LinRegPCR.nl.

### *JAK2* exon 9 PCR: normalisation for *JAK2* copy number

Normalisation of the *JAK2* copy number is required for accurately creating a dilution series. We amplified an 85 bp amplicon from exon 9 of the *JAK2* gene (GenBank accession number EF194100). Reactions were carried out in white 96 well LightCycler 480 Multiwell Plates on the LightCycler 480 (Roche Diagnostics, Penzberg, Germany) in a 10 μl final reaction volume comprising 1X PCR Buffer (Qiagen) containing 1.5 mmol/L of MgCl_2_, 200 μmol/L of each deoxynucleotide triphosphate (Fisher Biotec, Perth, Australia), 400 nmol/L of the forward primer and 200 nmol/L of the reverse primer (*JAK2_Ex9_F*: 5′-TTAACTGCAGATGCACATCATTACCT-3′ and *JAK2_Ex9_R*: 5′-GGCCATGACAGTTGCTTTGTATATT-3′) (GeneWorks, Adelaide, Australia), 5 μmol/L of SYTO 9 (Invitrogen, Carlsbad, CA), 0.25 units of HotStar Taq DNA polymerase (Qiagen).

2 μl of template was used at a concentration of 16.5 ng/μl, which corresponded to a total of about 10,000 *JAK2* copies per reaction.

The PCR conditions included an initial denaturation of 15 minutes at 95°C, followed by 60 cycles of 20 seconds at 94°C (when run along with the V617F mutation assay; 55 cycles when run by itself for quantification), 40 seconds at 63°C, 30 seconds at 72°C; 1 cycle of 1 minute at 95°C, 1 minute at 45°C, and a high resolution melting (HRM) step from 65°C to 95°C, increasing at 0.2°C per second. The HRM step was included as a quality control step to identify non-specific amplification.

Both the 100% mutant and the 100% wild-type controls were normalised for *JAK2* copy number in order to begin with equivalent numbers of amplifiable *JAK2* templates. The two cell lines were normalised against DNA obtained from a normal peripheral blood sample which was used as Cq reference based on its high quality and diploidy. LinRegPCR 12.5 software was used to determine the Cq values obtained from the normal peripheral blood DNA and from the HEL and HL-60 DNA control samples. The resulting values were used to adjust the concentration of each DNA, so that each had equivalent numbers of amplifiable *JAK2* templates.

### QuanTAS-PCR

The quantitative PCR method developed in this study consists of two PCR assays; a mutation-specific assay and an exon 9 copy number normalising reference assay (as already described above). For the detection of the *JAK2* exon 14 V617F mutation, the mutant allele-specific competitive blocker assay amplifies only the mutant allele. The *JAK2* V617F mutation PCR assay was designed complementary to the sense strand for the region framing the V617F mutation on *JAK2* exon 14. It consists of three oligonucleotides. The first is a mutant allele-specific forward primer *JAK2_Ex14_Mut_F*: 5′-CTTACTCTCGTCTCCACAGA**A**-3′ (where the bold “A” marks the position of the V617F nucleotide substitution). The second is the reverse primer *JAK2_Ex14_R*: 5′-TTCCTTAGTCTTTCTTTGAAGCAG-3′ resulting in an amplicon that is 101 bp in length. The third is a blocker oligonucleotide with a dideoxycytidine (ddC) at its 3′ end: *JAK2_Ex14_WT_Blocker_F*: 5′-CTTACTCTCGTCTCCACAGA-**ddC**-3′ (Sigma Aldrich, St. Louis, MO, USA).

**Figure 1:**
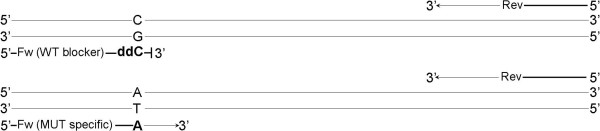
**Representation of the mutant allele-specific PCR and 3′ dideoxy blocker oligonucleotide methodology.** Two PCR primers, a mutant allele-specific forward primer and a reverse primer, are present in each reaction. In addition, a forward wild-type allele-specific dideoxy blocker (designated WT blocker), which binds to the wild-type allele but is incapable of extension, is present. It will outcompete the forward mutant allele-specific primer (designated MUT specific) for binding to the wild-type template and, coupled with the already low rate of amplification of a mutant allele-specific primer from a wild-type template, will further reduce the rate of false-positive amplification.

The dideoxy blocker competes with the mutant allele-specific forward primer and preferentially anneals to wild-type templates. Extension of the 3′ dideoxy oligonucleotide is not possible. The dideoxy blocker suppresses false-positive amplification by outcompeting the forward mutant allele-specific primer for binding to the wild-type template (Figure 1).

The *JAK2* mutation-specific PCR reactions were performed in a 10 μl final volume comprising 1X PCR Buffer containing 1.5 mmol/L of MgCl_2_, 0.5 mmol/L of extra MgCl_2_ (for a final concentration of 2 mmol/L of MgCl_2_), 200 μmol/L of each deoxynucleotide triphosphate, 200 nmol/L of the *JAK2_Ex14_WT_Blocker_F* oligo, 400 nmol/L of the *JAK2_Ex14_Mut_F* primer, 200 nmol/L of the *JAK2_Ex14_R* primer, 5 μmol/L of SYTO9, 1X Q-Solution (Qiagen), 0.25 units of HotStar Taq DNA polymerase and 2 μl of template (at a concentration of 16.5 ng/μl), for a total of about 10,000 *JAK2* copies per reaction. Early experiments did not include the Q-Solution PCR specificity enhancer.

The *JAK2* exon 9 PCR was used as a reference assay since it amplifies every *JAK2* allele present in the reaction. The *JAK2* exon 9 PCR was run along with each *JAK2* V617F mutation-specific PCR, in order to normalise each sample for its input number of *JAK2* templates, thus enabling accurate quantification of *JAK2* mutant templates.

Reactions were carried out in white 96 or 384 well LightCycler 480 Multiwell Plates on the LightCycler 480 and both PCR assays were run simultaneously under the following optimised conditions: an initial denaturation of 15 minutes at 95°C, followed by 60 cycles of 20 seconds at 94°C, 40 seconds at 63°C, 30 seconds at 72°C; 1 cycle of 1 minute at 95°C, 1 minute at 45°C and a HRM step from 65°C to 95°C increasing at 0.2°C per second. The HRM step was included as a quality control step to identify non-specific amplification.

For the *JAK2* exon 9 reference assay all samples were run in duplicate or triplicate (depending on the total amount of DNA available for each sample). We used 33 ng of DNA per reaction, which corresponds to 10,000 *JAK2* copies/well in the case of a high quality DNA preparation. (It is important to note that for low-quality/degraded DNA samples, more material would need to be applied to reach the same total *JAK2* copy number, as the amplifiable templates will be fewer in number. This can be monitored using the *JAK2* exon 9 reference assay and comparing the Cq of any high quality diploid genomic DNA preparation to the Cq of the low-quality/degraded samples tested.)

Samples were run in triplicate for the *JAK2* mutation-specific PCR. If any of the 3 replicates showed no amplification, we repeated the assay by running 10 replicates of that specific sample. The low-mutation-level mixes (MUT/WT 0.03% and 0.01%) were always run in replicates of 10. Using an increased number of replicates for the low concentration samples firstly allowed us to have a reliable reference for the Cq value obtainable from one single copy of the mutant allele and secondly, produced an increased amount of data which facilitated the use of a statistics-based approach for the estimation of the average mutant *JAK2* copy number (see below). The negative control samples (genomic DNA from healthy blood donors and cell lines DNA samples) were run in at least 10 replicates.

### Real-time data analysis

The real-time PCR data were analysed using LinRegPCR 12.5 software. The raw run data (not-baseline-corrected) for both the *JAK2* exon 9 PCR (reference assay) and the *JAK2* mutation-specific PCR were transferred from the LightCycler 480 to the LinRegPCR 12.5 software using the “LC480 Conversion: conversion of raw LC480 data” software (available on the Heart Failure Research Center website, at http://downloads.hfrc.nl, and treated as two different data sets (amplicon groups).

The LinRegPCR program performs sample by sample baseline correction, and finds a “window-of-linearity” for each amplicon group. This software uses linear regression to fit a straight line through each set of amplification curves: the slope of this regression line gives the PCR efficiency for each individual sample. It also estimates the “mean PCR efficiency” (*E*) for each amplicon by calculating the mean value of all sample efficiencies obtained per amplicon group. In performing the mean efficiency per amplicon calculation, the software was manually set to automatically exclude efficiency values deviating more than 5% from the median efficiency and samples which did not show amplification.

The data were first analysed using the automated LinRegPCR functions and subsequently manually corrected where required (as suggested by the LinRegPCR user manual, version 12.x). The fluorescence quantification threshold (*Nq*) was set to “common” for the *JAK2* exon 9 and *JAK2* exon 14 amplicon groups.

The LinRegPCR software uses the *Nq* value and the fractional cycle number (*Cq*) (needed for each sample to reach the *Nq* threshold) to calculate the starting concentration (*N*_*0*_) of each target (expressed in arbitrary fluorescence units), according to the equation *N*_*0*_ *= Nq/E*^*Cq*^*.* The *N*_*0*_ values obtained for each sample and for the 100% mutant control (HEL DNA)*,* for both the *JAK2* exon 9 control assay and the *JAK2* exon 14 test assay are then used to calculate the *JAK2* mutant load for each of the sample replicates, as described by the equation:

$$\;\text{Mut}\;\%\;=\frac{\frac{\left(\text{Ex}14\right)\;\text{Individual}\;\text{sample}\;\text{replicates}\;N0\;\text{value}}{\left(\text{Ex}9\right)\;\text{Average}\;\text{sample}\;\text{replicates}\;N0\;\text{value}}}{\;\frac{\left(\text{Ex}14\right)\;\text{Average}\;100\%\text{Mutant}\;\text{replicates}\;N0\;\text{value}\;}{\left(\text{Ex}9\right)\;\text{Average}\;100\%\text{Mutant}\;\text{replicates}\;N0\;\text{value}\;}}\times 100$$

The numerator in this formula measures the mutant (exon 14) to total (exon 9) *JAK2* ratio for a given sample, and the denominator normalises this value by calculating the same ratio for the 100% *JAK2* mutant control.

The formula allowes the monitoring of possible variations within the resulting mutation burden values of the replicates of each sample. The mean *JAK2* mutant-allele burden per sample was calculated by averaging the individual percentage for each replicate of that sample, excluding any obvious outlier values.

### Criteria to minimise the scoring of false positives

We then aimed to develop criteria to minimise the scoring of false positives. For this, it was necessary to identify the Cq value given by the amplification of a single mutant allele. Running 10 replicates of the 0.01% MUT/WT mix in each PCR run allowed us to have a reliable reference for the Cq value obtainable from one single copy of the mutant allele, which was then used as a guideline for the identification of rare false-positive occurrences. Signals that amplify significantly later are considered to be false positives, as it is not possible to have a template number between 1 and 0.

### Samples with very low numbers of mutant alleles

Samples with very low numbers of mutant alleles presented a particular challenge for quantification. In these samples, there will be no specific amplification in one or more of the replicates due to stochastic variation in the number of mutant alleles present per well. For example, if the average number of copies per replicate is 1 (i.e 1 mutant allele in the typically 10,000 alleles tested per well), there may be 0, 1, 2, or rarely more than 2 copies in a given replicate (in accordance with Poisson distribution).

**Figure 2:**
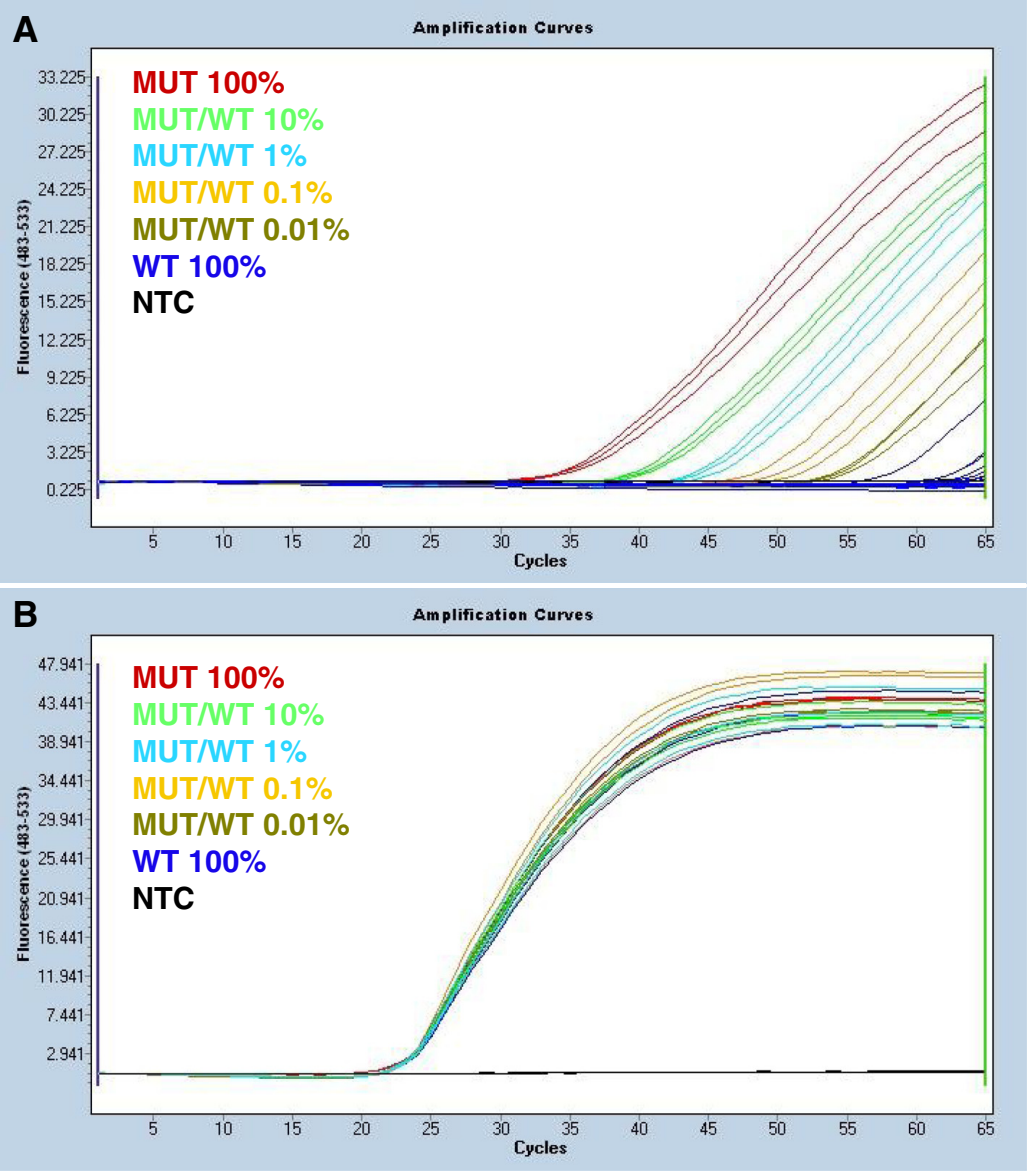
**The sensitivity of QuanTAS-PCR for quantifying *JAK2 *mutant alleles.** The *JAK2* mutation-specific qPCR shown in panel A and the *JAK2* exon 9 reference qPCR shown in panel B were run simultaneously (raw data (not baseline corrected) are shown). A range of samples containing different percentages of mutant allele in a background of wild-type allele were tested. For simplicity, only selected dilutions of the entire standard curve (prepared as described in the Methods) are shown. **A:***JAK2* mutation-specific PCR. The 100% mutant control DNA (MUT 100%), the mutant in wild-type mixes (MUT/WT) 10%, 1% and 0.1% and the no template control (NTC) were run in triplicate. The mutant in wild-type mix MUT/WT 0.01% and the 100% wild-type control (WT 100%) were run in 10 replicates. One of the 10 WT 100% replicates (in blue colour) amplified very late (LinRegPCR-calculated *Cq* = 58.2) compared to a single copy at 56.2. This represents a typical false positive and the sample was considered negative for the V617F mutation. The NTC did not amplify, as expected. **B:***JAK2* exon 9 reference qPCR used for DNA input and normalisation. Each sample was run in triplicate. The graph shows that all samples had an equivalent number of *JAK2* templates. The NTC did not amplify, as expected.

For low copy number replicates which show multiple wells with no amplification, it is possible to determine when a single mutant *JAK2* copy has been amplified (Figure 2). A single copy will have the lowest possible Cq value that is achievable for a true positive. Two copies will amplify earlier by a Cq value that is proportional to the amplification efficiency of the assay. As our *JAK2* mutation-specific assay typically has an amplification efficiency approximately equal to 1.7, this will be close to 1 Cq earlier. The probabilities of occurrence of each of the counts, y, can be modeled as a modified version of the Poisson distribution (right-censored Poisson distribution) with mean copy number, λ. The possible outcomes for more than two copies are grouped together as a single observation type, as we are unable to resolve these further. The probabilities for the number of *JAK2* V617F copies in a given experiment are calculated as described by the formula:

$$\;\text{Pr}\left(y;\lambda \right)=\left\{\begin{array}{cc} \frac{\lambda^{y}e^{-\lambda }}{y!}\;, & y\leq 2\\ 1-e^{-\lambda }\left(1+\lambda +\frac{\lambda^{2}}{2}\right)\;, & y>2 \end{array}\right)$$

The value of lambda (λ) represents the average copy number of mutant *JAK2* alleles assessed per replicate (e.g. the standard curve mix MUT/WT 0.03% will contain an average copy number λ = 3, when testing 10,000 *JAK2* copies per tube). The formula consists of two components. The first component (y ≤ 2) describes the Poisson distribution probability of observing y = 0, 1 or 2 copies of mutant *JAK2*. The second component (y > 2) describes the Poisson distribution probability of observing more than 2 copies of mutant *JAK2*. The probability of observing more than 2 copies is given as one minus the probabilities of observing 0, 1 and 2 copies.

A point estimate of the average number of mutant *JAK2* copies was obtained as the value of λ that maximises the likelihood of this distribution, given the observed number (y) of mutant *JAK2* copies in each experimental replicate (see formula above). The associated 95% confidence interval was obtained finding the values of λ for which the application of the likelihood test would not reject the null hypothesis of λ ≠ λ_0_, where λ_0_ is the value lambda found by the maximum likelihood-based method described above. These calculations were performed using the R statistical computing software v2.141.0 [31]. The relevant code is provided (Additional file 1).

## Results

### The use of high resolution melting (HRM) as a quality control step

The exon 9 and the *JAK2* V617F mutation-specific PCR assays use probe-free real-time amplification with a fluorescent intercalating dye (SYTO 9) to enable both quantification and HRM. The dye binds to double stranded DNA specifically and fluoresces only when intercalated. The HRM step allows the ready identification of any non-specific amplification, such as primer dimers or non-targeted sequences, both during optimisation experiments and routinely as a quality control for all experiments. Use of HRM in preference to gel electrophoresis both improves the workflow and eliminates PCR product manipulation as HRM is performed in the same reaction vessel immediately after the real-time amplification (often described as a closed-tube system).

**Figure 3:**
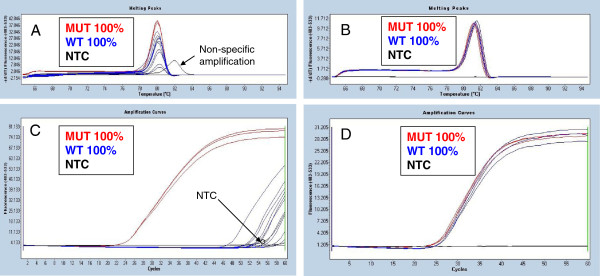
**HRM as a quality control step.** The use of an intercalating dye rather than a fluorescent probe to monitor the reaction allows the addition of an HRM step to identify non-specific amplification. Samples tested: 100% mutant control DNA (MUT 100%) analysed in triplicate, 100% wild-type control DNA (WT 100%) analysed in 10 replicates, non-template control (NTC) analysed in triplicate. **A**. *JAK2* V617F mutation-specific PCR high resolution melting. The NTC amplification product melted at a temperature clearly different (higher) than that of the targeted sequence, showing this was a case of non-specific amplification. It is important to note that the WT 100% amplification deriving from non-specific annealing of the mutant-specific forward primer to wild-type templates cannot be distinguished by HRM. **B**. *JAK2* exon 9 PCR high resolution melting. All PCR products show the same melting profile, indicating no presence of non-specific amplification products. **C**. *JAK2* V617F mutation-specific PCR amplification (early experiment, no Q-Solution was used). One of the NTC replicates amplified at a Cq value of about 54.6 and multiple WT 100% replicates amplified at Cq values that ranged from 46.5 to 58. **D**. *JAK2* exon 9 PCR amplification, performed to check for DNA input and for normalisation.

While we did not see primer dimers, non-specific amplification was occasionally seen during the optimisation experiments. Figure 3 (panel A) shows an example of non-specific amplification. It should however be noted that false-positive amplification due to mutant primers binding to wild-type templates will not be differentiated from true positive amplification by means of HRM analysis.

### The use of a 3′ dideoxy blocker

**Figure 4:**
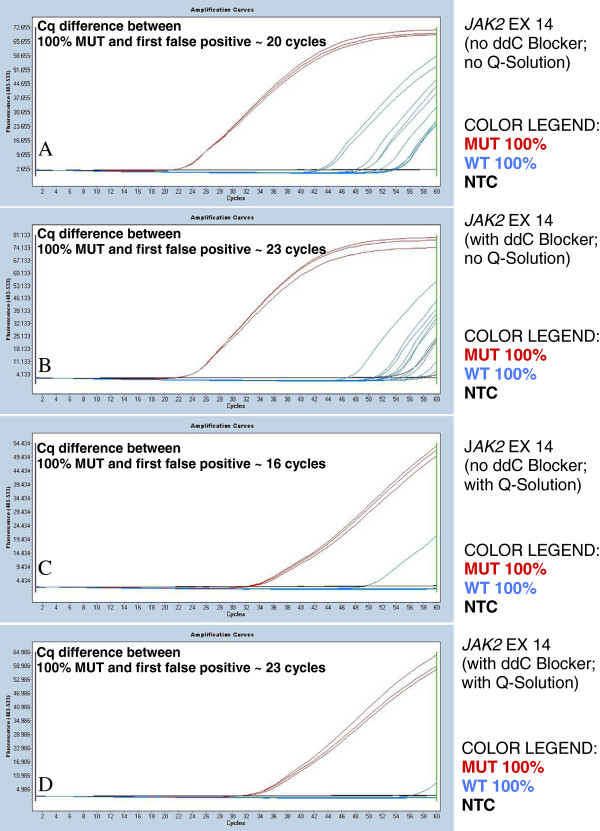
**Increasing the assay specificity of the *JAK2 *V617F mutation-specific PCR.** Samples tested: 100% mutant control DNA (MUT 100%) analysed in triplicate, 100% wild-type control DNA (WT 100%) analysed in 10 replicates, non-template control (NTC) analysed in triplicate. **A**. Mutant allele-specific PCR. The reactions contained two oligonucleotides: the mutant allele-specific forward primer and the reverse primer. The graph shows a significant number of false-positive amplifications. We observed a Cq value difference of 20 cycles between the MUT 100% and the first false-positive amplification. **B**. Mutant allele-specific PCR with the introduction of the wild-type specific 3′ dideoxy blocker. The reactions contained three oligonucleotides: the mutant allele-specific forward primer, the wild-type allele specific blocker and the reverse primer. The graph still shows the presence of a number of false positives. We observed a Cq value difference of 23 cycles between the MUT 100% and the first false-positive amplification. **C**. Mutant allele-specific PCR with the introduction of 1X Q-Solution. The reactions contained two oligonucleotides: the mutant allele-specific forward and the reverse primers. The graph shows a significant reduction of false-positive amplifications to a single false positive. We observed a Cq value difference of 16 cycles between the MUT 100% and the first false-positive amplification. **D**. Mutant allele-specific PCR with the introduction of both the 3′ dideoxy blocker and 1X Q-Solution. The reactions contained three oligonucleotides: the mutant allele-specific forward primer, the wild-type allele-specific blocker and the reverse primer. One false-positive amplification was observed, at a very late Cq value. We observed a Cq value of 23 cycles difference between the MUT 100% and the first false-positive amplification.

The 100% mutant and 100% wild-type controls were normalised to the same amplifiable *JAK2* copy number using the exon 9 assay. Significant false-positive amplification from the wild-type template was observed without the 3′ dideoxy blocker (see Figure 4; panel A). With the dideoxy blocker, there was an improvement but still considerable false-positive amplification (see Figure 4; panel B). Although the introduction of the dideoxy blocker might have been expected to completely suppress false-positive amplification from the wild-type template, the false-positive amplification still detected was probably due to the occasional annealing of the mutation-specific forward primer to wild-type templates. Increasing the concentrations of the dideoxy blocker did not improve the overall assay results.

### Increased specificity with the use of Q-Solution

We tested the effect of using the proprietary PCR specificity enhancer Q-Solution (Qiagen) in addition to the dideoxy blocker to test whether we could further reduce false-positive amplification from wild-type templates. Q-Solution was added to the *JAK2* mutation-specific PCR, both with and without the dideoxy blocker. We found that the use of Q-Solution alone increased the analytical specificity of the assay, reducing the early occurrence of false positives, possibly by decreasing the chance of false-priming. Nevertheless, we found that false positives could (very rarely) occur at relatively early Cq values with the use of Q-Solution alone (see Figure 4; panel C for an example of this). When we added the dideoxy blocker to the Q-Solution, we observed an even greater Cq value differential between the controls and the false positives (see Figure 4; panel D). Therefore, the combined use of the 3′ dideoxy blocker and Q-Solution in QuanTAS-PCR was our chosen approach for reducing false positives. However, it should be noted again that false positives are very rare in both Q-Solution only conditions and in Q-Solution with dideoxy blocker conditions, and thus the Cq differential may not be an accurate guide to the differences between the assays.

The addition of Q-Solution resulted in a decrease in amplification efficiency, which can be explained by a shift of the annealing equilibrium between primers and template. The loss of PCR efficiency and the consequent increase in the Cq values of all the amplified samples did not impair the assay’s analytical sensitivity. Similar results were obtained with betaine which is likely to be the principal component of Q-Solution, but this was not explored further.

### Optimisation of DNA input

In order to design a quantitative PCR assay that could comfortably detect a very low mutant to wild-type ratio, we established the optimum amount of the DNA required. This was to avoid high amounts of genomic DNA resulting in PCR inhibition or strong background noise, while keeping the template number as high as possible. We carried out a comparative assay, analysing the different sensitivities obtained when 33, 66, 99 or 198 ng/well of DNA were used (corresponding respectively to 10,000 *JAK2* copies/well, to 20,000 *JAK2* copies/well, to 30,000 *JAK2* copies/well and to 60,000 JAK2 copies/well).

The detection of mutant alleles at a sensitivity of one mutant allele copy per replicate was best achieved when testing 33 ng of DNA per well (corresponding to 10,000 *JAK2* copies/well). The results were concordant, both with and without the addition of Q-Solution to the reactions containing the dideoxy blocker. At higher DNA amounts per well, we observed a proportional increase in background noise in the amplification curves which were not quantifiable using the LinRegPCR software, and did not improve the assay sensitivity. No significant background noise was observed when 33 ng of DNA were used, enabling the LinRegPCR analysis to be readily performed (Additional file 2).

### Criteria for the identification of false positives

Visual examination of replicates of the 0.01% and 0.03% MUT/WT mixes in each run allowed us to identify the Cq value of a single copy of the mutant allele, which was then used as a guideline for the identification of rare false positives. Samples that amplified 2 or more cycles later than a single mutant allele were considered to be false positives, as it is not possible to have amplification of less than one template.

### Testing of negative controls

A total number of 13 “negative” control DNA samples from randomly selected, healthy blood donors were tested. Each sample was confirmed to be operationally negative for the *JAK2* V617F mutation by testing 10 replicates. Each showed no positives in all replicates when assessed with QuanTAS-PCR. Occasionally we observed one replicate out of the 10 amplifying at a very late Cq value, significantly later than the Cq value for the amplification of a single mutant allele copy.

In order to generate a standard series of controls, a *JAK2* V617F mutation-negative cell line was required. We chose the HL-60 cell line, given that this cell line (and the V617F mutant allele positive HEL cell line) are readily available. We tested 60 replicates of HL-60 DNA using QuanTAS-PCR at the optimised conditions to verify that it was truly negative for the mutation. Only 1 replicate out of 60 showed any amplification. However, this was at a LinRegPCR-calculated Cq value equal to 58.2 (Figure 2; panel A)*,* whereas on the same PCR run, one single copy amplified at a LinRegPCR-calculated Cq value of 56.2, and thus could be easily determined to be an artefactual false positive. These results were confirmed by analysing our data using the maximum likelihood-based method (described in the Methods). According to the statistical analysis, the WT 100% DNA extracted from the HL-60 cell line was estimated to have an average *JAK2* copy number of 0.016 (confidence interval 0.001 - 0.073) per reaction input. Since this value is considerably less than one, we can confidently regard such late amplification as a false positive. Thus HL-60 was confirmed to be a suitable 100% wild-type control.

### Analytical sensitivity

In order to assess the analytical sensitivity of the QuanTAS-PCR assay, a set of mutant/wild-type mixes were prepared by mixing Cq normalised HEL DNA (MUT) and HL-60 DNA (WT). Samples with the *JAK2* V617F mutant allele present at levels of 100%, 30%, 10%, 3%, 1%, 0.3%, 0.1%, 0.03%, 0.01% were tested as well as the 100% wild-type control. The assay showed an analytical sensitivity of one mutant allele per well (Figure 2; panel A).

When testing the 0.03% and 0.01% mixes (ten replicates each), we also calculated the average *JAK2* copy number for each replicate. We obtained a value of 2.00 copies (confidence interval 1.20 - 3.10) for the 0.03% mix and a value of 0.60 (confidence interval 0.24 - 1.22) for the 0.01% mix. Both estimations can be considered sufficiently accurate, when compared to their corresponding expected values equal to 3 and 1 allele copies per well respectively.

### Validation of mutant allele quantification in clinical samples

DNA from 38 patients that had previously been tested by the less sensitive ACB-PCR assay [17] was tested in a blinded fashion using QuanTAS-PCR at the optimised conditions. As expected from the analytical sensitivity experiments, QuanTAS-PCR identified more positive samples than the qualitative ACB-PCR assay (Additional file 3). Five samples that were scored as negative for the V617F mutation by the ACB-PCR assay were found to be positive when tested with the QuanTAS-PCR, with calculated mutant allele burdens that ranged from 2.03% to 0.13% (namely 2.03%, 0.27%, 0.23%, 0.13% and 0.13%). QuanTAS-PCR showed improved repeatability, showing highly consistent Cq values within triplicate reactions. In contrast, ACB-PCR showed a mix of signal intensities within the replicates of seven samples, with some replicates being strongly positive and others weakly positive or even negative for the V617F mutation when PCR products were resolved on an agarose gel (Additional file 3).

**Figure 5:**
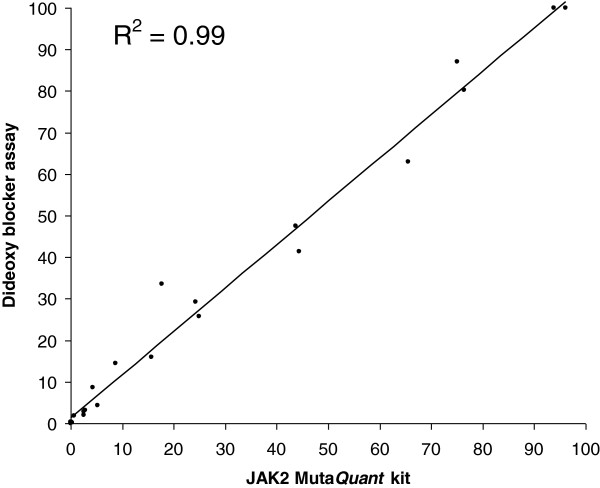
**Regression analysis of the mutant allelic percentages of 24 clinical samples determined using the “JAK2 Muta*Quant*” assay (Ipsogen) (on the x axis) and QuanTAS-PCR assay (on the y axis).**

The *JAK2* mutation levels of 24 selected samples (all of which had tested positive for the mutation using QuanTAS-PCR) were also tested with the commercial quantitative kit “JAK2 Muta*Quant*” (Ipsogen SA, Marseille, France). The two assays showed comparable results, with a coefficient of determination (R^2^) equal to 0.99 (Figure 5). QuanTAS-PCR scored two samples as having averaged mutant allele burden values equal to 113.7% and 148.7%. Values greater than 100% can occur occasionally with normalised real-time PCR data. In these two cases we scored and plotted the samples as 100% mutant (Additional file 3).

## Discussion

Whereas for diagnostic purposes, an analytical sensitivity of 1% is generally sufficient, minimal residual disease studies require greater sensitivity, specificity and accurate quantification. Many *JAK2* V617F mutation detection methodologies have been developed and published over the last few years, only some of which are applicable to minimal residual disease [7-11,13-18,21-27].

A recent multicenter study established that only two of the eleven tested methods, both based on TaqMan allele-specific qPCR, could reliably achieve an analytical sensitivity of 0.2% [32]. However, it was more recently reported that these two assays have problems with false-positive results, whereas an alternative Locked Nucleic Acid (LNA) based qPCR method showed a sensitivity of 0.4% [22].

A two step PCR method that claimed an analytical sensitivity of 0.001% was recently reported [27]. The first step enriched the mutant allele fraction using a LNA-based wild-type blocking system, whilst the subsequent PCR step was an allele-specific qPCR to quantify the mutant allele. The claimed sensitivity is unlikely as the sensitivity of any methodology is limited by the number of templates initially used in the assay. This sensitivity could only be reached beginning with 100,000 templates or 300 ng of DNA in the tube. In addition, the first enrichment step will prevent this method from being truly quantitative. Furthermore, any two step approach (even one which has a limited number of amplification cycles in the first step) increases the risk of cross-contamination between samples when performing the second step.

We developed a novel single-tube, single-step, mutant allele-specific quantitative PCR assay: QuanTAS-PCR (Quantitative Threefold Allele-Specific PCR) assay. It uses a combination of established and novel features. We combined the basic principles of (i) the use of a 3′mismatched primer in allele-specific PCR, [33], (ii) of a wild-type blocker [34] and (iii) we introduced the use of a PCR specificity enhancer.

In allele-specific PCR, the 3′ end of the mutant allele-specific primer is placed over the mutation site, so that mutant templates are preferentially amplified [35]. However, as numerous investigators have found, extensions from the wild-type allele do still occur at low frequencies. Mutant-specific amplification is enhanced by the addition of a wild-type blocker to the PCR reaction [34]. A wild-type blocker is an oligonucleotide complementary to the wild-type allele with its 3′ end placed over the mutation site. The blocker also bears a 3′ modification which is incapable of 3′-5′ extension, therefore “blocking” the wild-type allele amplification. In our assay, we used a 3′dideoxycytidine oligonucleotide blocker complementary to the sense strand where the wild-type sequence terminates at the G at position 1849.

Dideoxy blockers are oligonucleotides bearing a dideoxy nucleotide (ddNTP) at their 3′ ends. Dideoxy nucleotides are effective chain terminators used in Sanger sequencing [36]. ddNTPs are essentially deoxyribonucleotides (dNTPs) without a 3′-hydroxyl group (-OH) on their deoxyribose sugar. Therefore 3′ddNTPs are unable to form a phosphodiester bond with any other dNTP and chain elongation is not possible.

A combination of allele-specific PCR and dideoxy oligonucleotide blockers was first reported for the analysis of disease causing mutations in several genes [37] but seems not to have been widely adopted*.* However, there are major differences between the previous methodology and the one reported here, including the use of qPCR rather than endpoint PCR.

We enhanced the analytical specificity of the mutation-specific assay by adding a proprietary co-solvent, Q-Solution, to the PCR reaction. This increased the PCR specificity of the *JAK2* mutation-specific assay, showing a further decreased occurrence of false-positive results when compared to the same assay run without Q-Solution. To the best of our knowledge, this is the first application of the use of a co-solvent to increase specificity for the detection of minimal residual disease. Importantly, the addition of a PCR specificity enhancer to the PCR reaction is more efficient in suppressing false-positive amplification from wild-type templates than the addition of a dideoxy blocker. By combining the use of a dideoxy blocker and a PCR specificity enhancer, we achieved high analytical sensitivity (one single mutant-allele per well) coupled with high analytical specificity, providing a robust quantitative assay.

Another unique feature of our methodology is the combined normalisation for the input of DNA and correction for copy number variation by calibrating directly to the *JAK2* copy number in a control PCR (*JAK2* exon 9 PCR), using efficiency-corrected calculation of the starting concentrations per sample. This reference PCR was used for each sample including the controls, allowing accurate quantification of the mutant allele burden.

The use of real-time PCR technology allowed us to distinguish false-positive amplifications from true positive ones, by determining the Cq value at which each sample replicate was amplified with the *JAK2* mutation-specific assay. We were able to readily identify false positives based on their high Cq values. False-positive samples are those that clearly amplify later than the reactions starting with a single copy of the template.

The quantitative *JAK2* exon 9 and mutation-specific PCR assays are designed to enable both real-time amplification analysis and HRM analysis in the one run on a real-time PCR instrument. SYTO-9, a fluorescent intercalating dye which does not inhibit PCR reactions when used at saturating conditions [38], binds double stranded DNA specifically and fluoresces only when intercalated. The use of a closed-tube method like the QuanTAS-PCR is desirable in a diagnostic setting, being quicker, more reliable, and able to minimise PCR carry-over problems.

When testing a sample that is expected to have very low levels of mutant *JAK2* alleles (below 0.03%), we recommend using additional replicates, and analysing the data using a statistical estimation approach such as the maximum likelihood-based method described in the Methods section. As a reference, 10 replicates of the 0.01% MUT/WT mix should be also run on each PCR run, in order to have a reliable indication of the Cq value of one single copy of the mutant allele. This Cq value can then be used as a cut-off to distinguish positive amplifications from any rare false-positive event.

Results obtained with QuanTAS-PCR were validated against those obtained with our previously published ACB-PCR qualitative assay [17]*.* The ACB-PCR assay was able to detect as few as 1% mutant alleles, but often presented some reproducibility problems, i.e. some sample replicates of the ACB-PCR assay showed ambiguous or even contrasting results between replicates. In these cases the ACB-PCR assay had to be repeated in order to confirm the positivity of those samples. Five clinical samples that were previously reported as negative for the V617F mutation when tested by ACB-PCR, were clearly positive when tested with the more sensitive QuanTAS-PCR, scoring mutant-allele burdens which ranged from 2.03% to 0.13%. Furthermore, the QuanTAS-PCR generated similar mutant-allele burden results for each of the sample triplicates, thus showing very good reproducibility (Additional file 3).

The QuanTAS-PCR’s quantitative results were also validated against those obtained with the commercially available kit “JAK2 Muta*Quant*” (Ipsogen) which is based on a dual TaqMan probe allelic discrimination approach. All of the samples that were determined to have the V617F mutation when tested with QuanTAS-PCR, were then tested in a blinded fashion with the Ipsogen kit. The two quantitative tests generated comparable mutant-allele burden values and the resulting values were very well correlated (Additional file 3).

## Conclusions

In this study, we have developed a mutant allele-specific quantitative PCR assay, with a unique combination of features; a mutation-specific primer, a 3′ dideoxy blocker, a PCR specificity enhancer and real time amplification analysis, which is suitable for minimal residual disease studies. A recent review concluded that a *JAK2* V617F detection assay should be both specific and sensitive enough to detect a mutant allele burden as low as 1–3% [28]. Thus QuanTAS-PCR is also suitable as a diagnostic method.

QuanTAS-PCR shows a very high analytical sensitivity and the ability to efficiently suppress wild-type allele amplification. We can consistently and reproducibly detect the presence of extremely low levels of *JAK2* V617F in a wild-type allele background, being able to detect as low as one mutant template per well. When higher numbers of replicates are tested, higher sensitivities are achievable depending on the total number of templates assessed. The combination of principles in this assay can thus be used in minimal residual disease monitoring, not just for *JAK2* V617F, but for other common recurrent single nucleotide mutations in solid tumours, e.g. *BRAF* c.1799T > A (V600E) or the acquired resistance mutation *EGFR* c.2369C > T (T790M).

## Abbreviations

ACB-PCR: Allele-specific competitive blocker PCR; ARMS: Amplification refractory mutation system; Cq: Quantification cycle; dNTPs: Deoxynucleotide triphosphate; HRM: High resolution melting; MPN: Myeloproliferative neoplasm; MUT: Mutant; NTC: No-template control; PCR: Polymerase chain reaction; WT: Wild-type.

## Competing interests

The authors declare that they have no competing interests.

## Authors’ contributions

GVZ co-designed the experimental concept, performed and validated the assays and co-wrote the manuscript. RNJ developed the low copy number analysis. CAH designed the reference assay. MM performed and provided the ACB-PCR results. DAW supervised the development of the assay and provided the clinical samples. AD co-designed the experimental concept, supervised the development of the assay and co-wrote the manuscript. All authors read and approved the final manuscript.

## Authors’ information

Alexander Dobrovic and David A Westerman are equal senior authors.

## Pre-publication history

The pre-publication history for this paper can be accessed here:

http://www.biomedcentral.com/1471-2407/13/206/prepub

## Supplementary Material

Additional file 1 **The R script used in determining average *JAK2 *mutant copy number.** Click here for file

Additional file 2 **LinRegPCR analysis: background noise increases as the DNA input per well increases.** The *JAK2* mutation-specific PCR amplification curves are shown without baseline correction. A range of samples containing different percentages of the mutant allele (MUT) relative to the wild-type allele (WT) were tested (MUT 100%, MUT/WT 30%, MUT/WT 10%, MUT/WT 3%, MUT/WT 1%, MUT/WT 0.3%, MUT/WT 0.1%, MUT/WT 0.03%, MUT/WT 0.01%). The four panels show how the background noise increases as the DNA input per well increases. Panel A: 33 ng of DNA per well; Panel B: 66 ng of DNA per well; Panel C: 99 ng of DNA per well. Panel D: 198 ng of DNA per well. The LinRegPCR software indicated that some of the samples could not be analysed due to high background noise when 66, 99 or 198 ng of DNA were tested, but this never occurred when using 33 ng DNA per well. As discussed in the Results section, we concluded that the optimal amount of DNA to be assessed per well corresponded to 33 ng (Panel A). Click here for file

Additional file 3 **Validation of QuanTAS-PCR *JAK2 *assay.** The results from the QuanTAS-PCR assay in comparison with the ACB-PCR assay and the Ipsogen JAK2 Muta*Quant* assay. Samples in grey were excluded from calculating the average score as they were obvious outliers. Click here for file
