# Supplementary material for: Quantitative threefold allele-specific PCR (QuanTAS-PCR) for highly sensitive JAK2 V617F mutant allele detection
Source: BMC Cancer. 2013 Apr 24;13:206. doi: 10.1186/1471-2407-13-206 (PMC3658971; doi:10.1186/1471-2407-13-206)
Supplement: Additional file 1 — The R script used in determining average JAK2 mutant copy number. [file 1471-2407-13-206-S1.doc]

The following R script can be used for the estimation of the average number of JAK2 mutant copies from a set of sample replicates, plus its associated 95% confidence interval, using the methodology described in this report. After downloading and installing the statistical computing program, R, one can simply copy and paste the commands shown below.

The line “y <- c(0, 0, 0, 0, 1, 1, 2, 2, 2, 3)” needs to be replaced with the numbers obtained from your data. The order of these data is unimportant. Note that the lines of text beginning with a hash symbol are comments which are ignored by R but provide some information to the user as to what the commands do. The final output from this script consists of the point estimate of the average mutant JAK2 copy number and the endpoints of its associated estimated 95% confidence interval.

# Your observed data (the order of the observations is not important)

y <- c(0, 0, 0, 0, 1, 1, 2, 2, 2, 3)

# Setting upper limit of the number of copies that can be resolved

k <- 2

# This function describes the modified Poisson distribution, as described in

# Zapparoli et al. It doesn't do anything until you call it

dpoisIneq <- function(x, lambda, k, log = FALSE){

ifelse(x <= k, dpois(x, lambda, log = log),

ppois(k, lambda, lower.tail = F, log = log)) }

# Define the function to calculate the likelihood (or log-likelihood)

# for a given value of lambda, given the data

# Aside: use coeff = -1 when using "optimize" for maximum likelihood

log.lik.dpoisIneq <- function(lambda, x, k, coeff = 1, offset = 0) {

coeff * sum(dpoisIneq(x, lambda, k, log = T)) + offset

}

# Plot the log-likelihood for your data. Make sure there is a distinct maximum

lmb <- seq(0.1, 5, 0.01)

ll.lmb <- sapply(lmb, log.lik.dpoisIneq, y, k)

plot(lmb, ll.lmb, type = "l", xlab = "lambda", ylab = "log-likelihood")

# Find the maximum likelihood estimate of lambda by optimizing the

# log-likelihood with respect to the data

r <- c(0, k * 5)

opt.log.lik <- optimize(log.lik.dpoisIneq, r, x = y, k = k, coeff = -1)

lambda.ML <- opt.log.lik$minimum

# Use a likelihood ratio-based approach to find the confidence limit endpoints

z <- qchisq(0.95, 1) # for 95% confidence interval

ofset <- opt.log.lik$objective + z / 2

root.lo <- uniroot(log.lik.dpoisIneq, c(0, lambda.ML),

x = y, k = k, offset = ofset)

ci.lo <- root.lo$root

root.hi <- uniroot(log.lik.dpoisIneq, c(lambda.ML, r[2]),

x = y, k = k, offset = ofset)

ci.hi <- root.hi$root

# Print final results

results <- c(lambda.ML, ci.lo, ci.hi)

names(results) <- c("Estimate", "ci.lo", "ci.hi")

print(results)
