# Supplementary figures and images for: Quantitative threefold allele-specific PCR (QuanTAS-PCR) for highly sensitive JAK2 V617F mutant allele detection
Source: BMC Cancer. 2013 Apr 24;13:206. doi: 10.1186/1471-2407-13-206 (PMC3658971; doi:10.1186/1471-2407-13-206)

## Slide 1
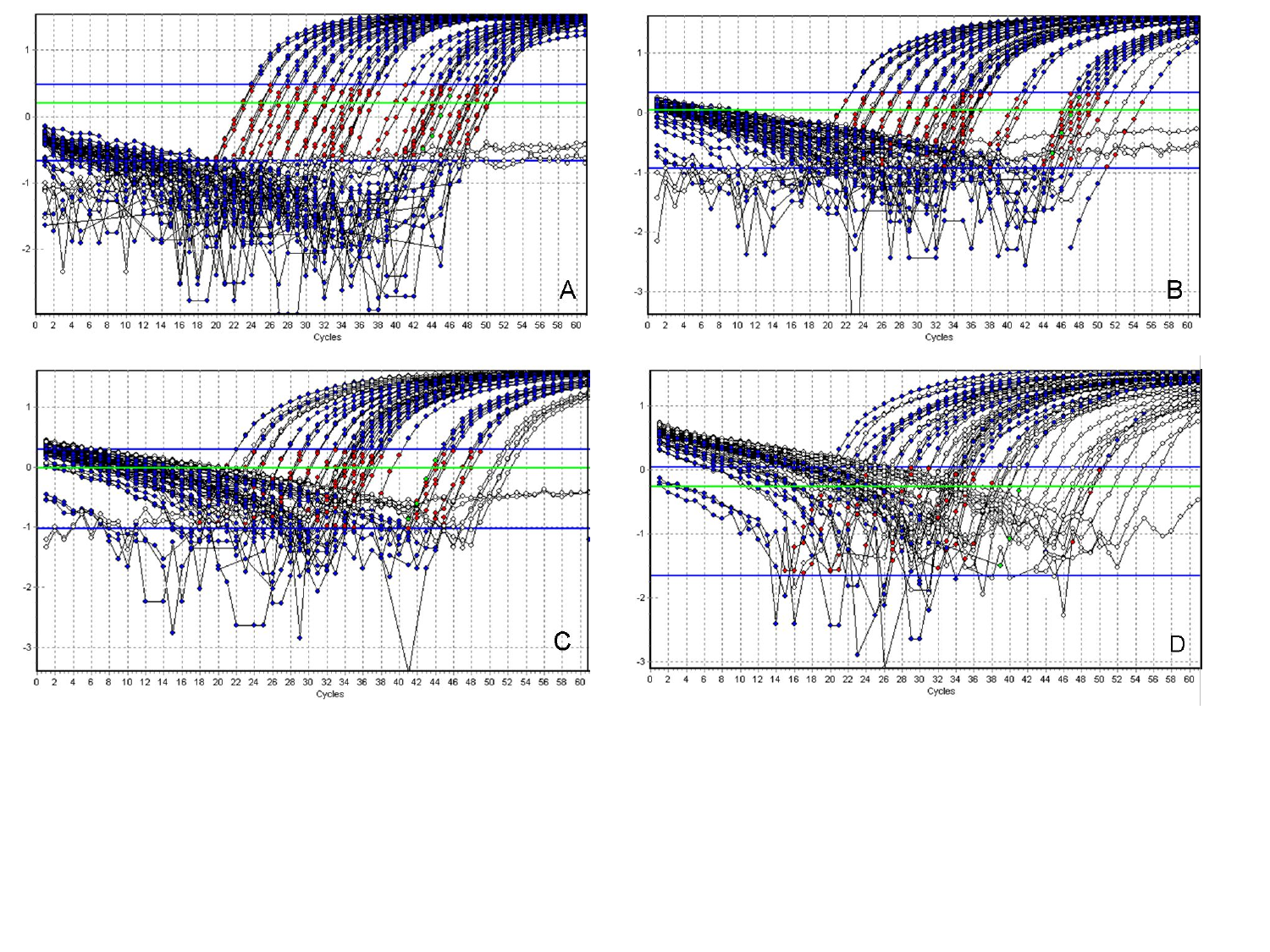

Supplement: Additional file 2 — LinRegPCR analysis: background noise increases as the DNA input per well increases. The JAK2 mutation-specific PCR amplification curves are shown without baseline correction. A range of samples containing different percentages of the mutant allele (MUT) relative to the wild-type allele (WT) were tested (MUT 100%, MUT/WT 30%, MUT/WT 10%, MUT/WT 3%, MUT/WT 1%, MUT/WT 0.3%, MUT/WT 0.1%, MUT/WT 0.03%, MUT/WT 0.01%). The four panels show how the background noise increases as the DNA input per well increases. Panel A: 33 ng of DNA per well; Panel B: 66 ng of DNA per well; Panel C: 99 ng of DNA per well. Panel D: 198 ng of DNA per well. The LinRegPCR software indicated that some of the samples could not be analysed due to high background noise when 66, 99 or 198 ng of DNA were tested, but this never occurred when using 33 ng DNA per well. As discussed in the Results section, we concluded that the optimal amount of DNA to be assessed per well corresponded to 33 ng (Panel A). [file 1471-2407-13-206-S2.ppt]
